# Supplementary figures and images for: Assessment of the sublingual microcirculation with the GlycoCheck system: Reproducibility and examination conditions
Source: PLoS One. 2020 Dec 23;15(12):e0243737. doi: 10.1371/journal.pone.0243737 (PMC7757800; doi:10.1371/journal.pone.0243737)

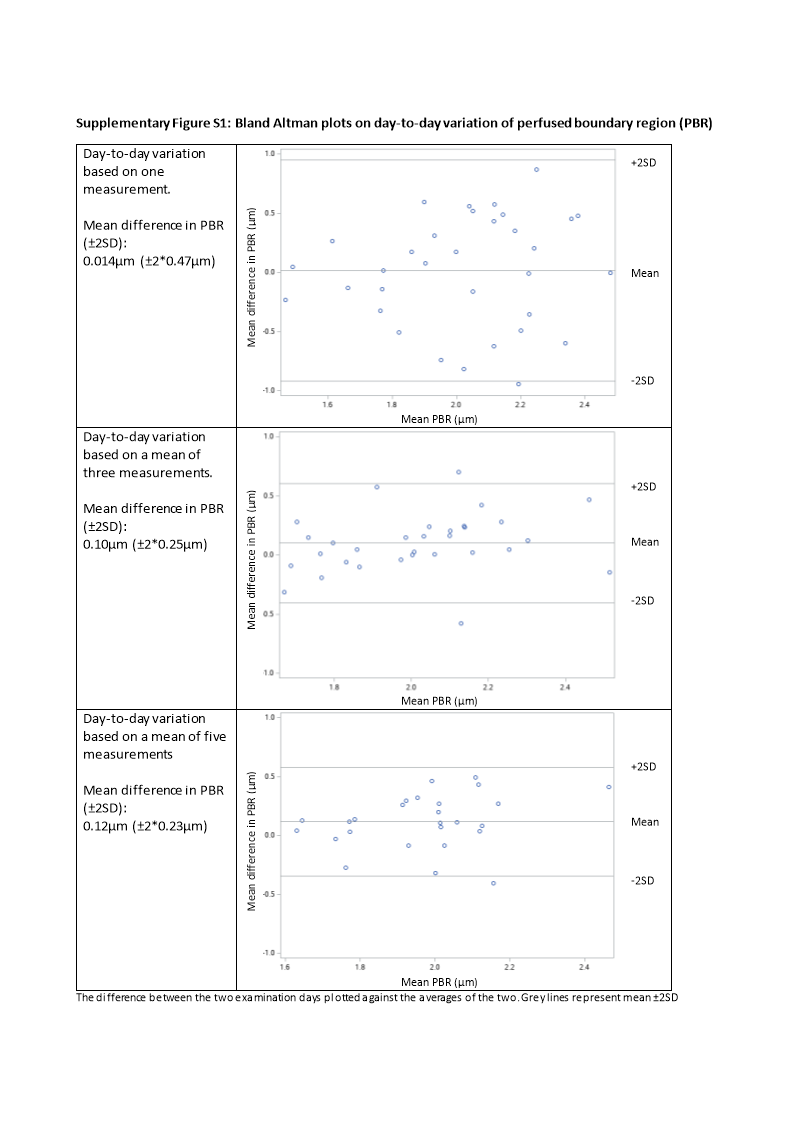

Supplement: S1 Fig — (TIF) [file pone.0243737.s001.tif]

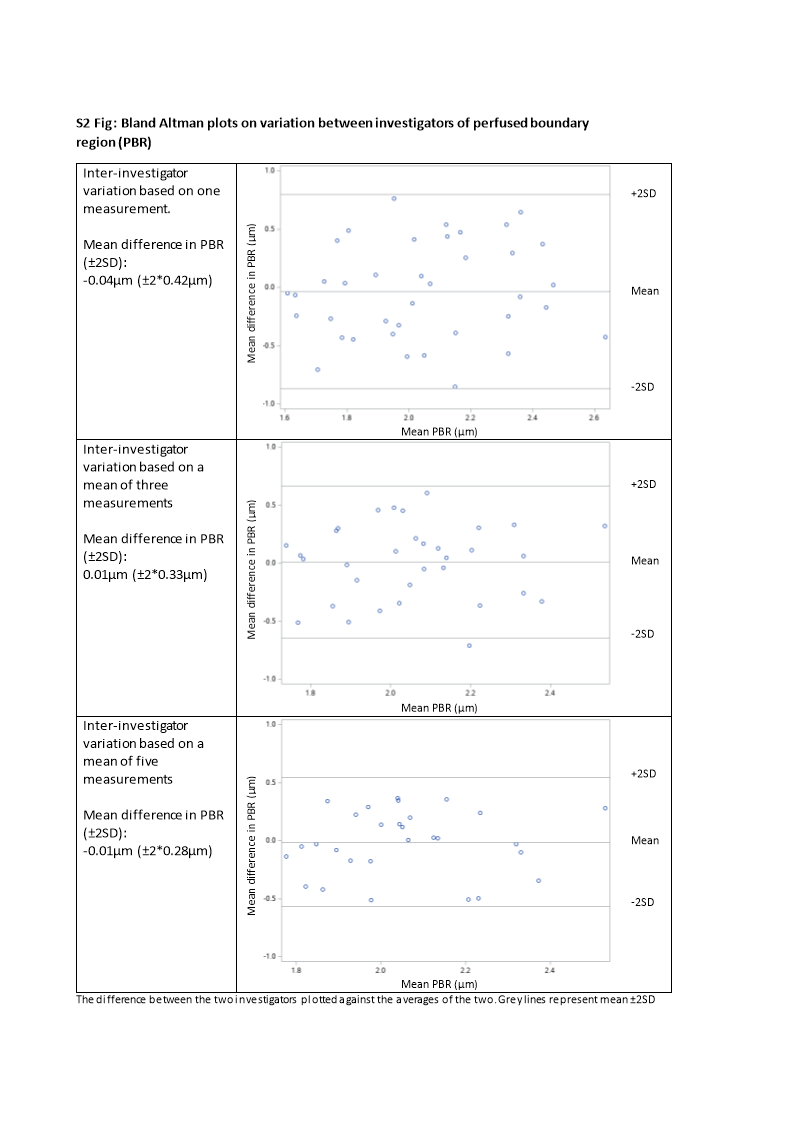

Supplement: S2 Fig — (TIF) [file pone.0243737.s002.tif]

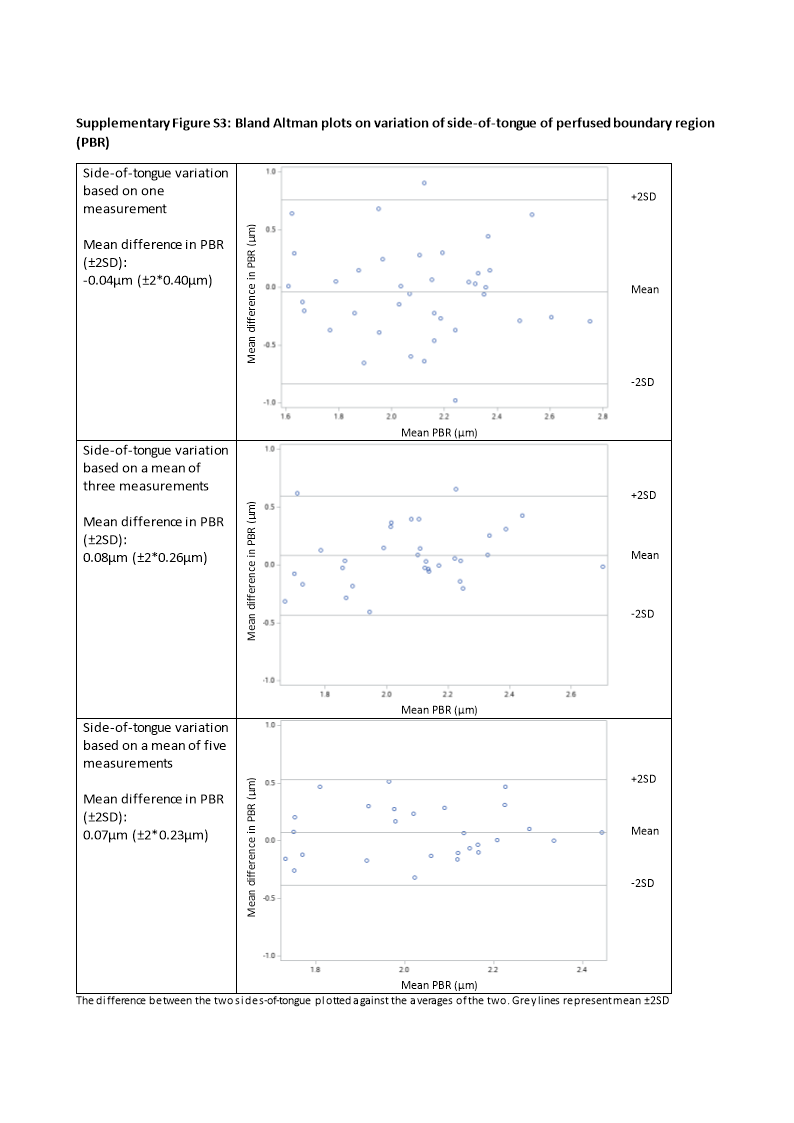

Supplement: S3 Fig — (TIF) [file pone.0243737.s003.tif]
