# Supplementary material for: Assessment of the sublingual microcirculation with the GlycoCheck system: Reproducibility and examination conditions
Source: PLoS One. 2020 Dec 23;15(12):e0243737. doi: 10.1371/journal.pone.0243737 (PMC7757800; doi:10.1371/journal.pone.0243737)
Supplement: Attachment — Submitted filename: Response to Reviewers MicroREx.docx [file pone.0243737.s004.docx]

**Answers to journal requirements and reviewers comments:**

**Journal Requirements:**

1. Please ensure that your manuscript meets PLOS ONE's style requirements, including those for file naming. The file names and headings are updated.

2.  You ask us to amend your current ethics statement to include the full name of the ethics committee/institutional review board(s) that approved your specific study. The full name is amended in the revised version.

You further ask us that once we have amended this/these statement(s) in the Methods section of the manuscript, we add the same text to the “Ethics Statement” field of the submission form (via “Edit Submission”). The submission form is updated accordantly.

3. In the online submission form you ask us to clarify whether any competing interests related to the manufacturer of the GlycoCheck System exist. This we have clarified.

4. You ask us to confirm that our conflicts of interest does not alter our adherence to all PLOS ONE policies on sharing data and materials, by including the following statement: "This does not alter our adherence to  PLOS ONE policies on sharing data and materials.” The statement is now included *(page 18 line 385)*.

You furthermore ask us to include our updated Competing Interests statement in our cover letter. This is updated.

5. Please include captions for your Supporting Information files at the end of your manuscript, and update any in-text citations to match accordingly. Please see our Supporting Information guidelines for more information: [http://journals.plos.org/plosone/s/supporting-information](about:blank).

*Thank you very much for drawing our attention to this. A list of supporting documents has been added at the end of the manuscript.*

**Reviewers' comments:**

Reviewer #1:


1) Please provide the figure displaying the capture of actual measurements with the device with labelled outer layer and PBR.

*Thank you for the suggestion. We appreciate the importance of a visualisation of the investigated recordings. We have included a representative image of the capillaries obtained with the GlycoCheck system in Figure 1.*

2) The authors state that multiple measurements should be performed to achieve reproducible measurements capable of detecting the effect by intervention. There was only slight improvement in ICC when conducting 5 measurements instead of 3. What would be the authors recommendation for standardization in this case?

*It is correct that an exact advice in this matter is warranted and we have included a statement that “at least three measurements would be advisable” in the Discussion page 14 line 280*

3) The authors investigated relatively young participants. Would there be an age-related effect on the accuracy of the glycocalyx measurements?

*Unfortunately we are not able to conclude on age effect within the design of this study. Therefore, statements in this matter would be speculations. We have included this as a limitation of this study: “As this is a study on young healthy individuals, we can only speculate on whether the accuracy of the measurements would be the same in groups which are different with regards to age or disease status.” (page 16 line 355).*

4) What is the effect of hydration on similar capillary measures? Did authors control for this effect?

*That is a very good point and it would have been great if that would have been possible. Unfortunately, we only measured haematocrit at baseline in this study and therefore we could not readily correct for hydration status as the change in haematocrit could not be measured. In the revised version we have calculated the association between the haematocrit and the capillary measures.:*

*“**The correlation between the haematocrit and the capillary measures at visit 1 was not significant (p>0.05)” (page 11, line 203). Furthermore, we added this point to limitations: “**We cannot exclude that changes in hydration status could impact the measures, but at baseline there was no association with haematocrit and PBR or RBC filling.” (page 17, line 359).*

5) The authors should discuss the potential effect of microvascular disease (T1D) or global vascular disease on these measurements.
*Thank you for this helpful suggestion which had led us to add to the Discussion: “Thus, a damaged glycocalyx measured locally could be viewed as an expression of generalised microvascular damage and possibly a precursor of complications as seen e.g. in diabetes with retinopathy, nephropathy, etc. which emphasises the importance of easy, non-invasive measurement.“ (page 13, line 251).*

Reviewer #2:

Introduction:
Page 4, line 64: The reference used is outdated (1966). Indeed, it is a challenge to measure the size of the glycocalyx in extracted tissues. However, nowadays this is possible with the use of different methods (PMID: 17256154 and atomic force microscopy PMID: 24278345, 24727235).

*It is indeed right that the used reference was outdated, and we have removed it. Instead we have included the suggest sentence, references and rephrased the paragraph:*

*“It has been a challenge to measure the size of the glycocalyx where measurements primarily have been performed invasively. However, nowadays this is possible with the use of different methods (15-17). A new non-invasive method has been developed named the GlycoCheck system - a handheld non-invasive camera (Capiscope handheld, KK Research technology Ltd) that is placed under the tongue to obtain in vivo video recordings of the blood flow in the capillaries coupled to the GlycoCheck software”. (page 4 Line 63).*

Page 4, line 67-68: I would kindly disagree with the authors at that point. Further studies have been conducted to evaluate the reproducibility of the PBR measurements (31743299, 29444696, 30764695). Moreover, besides reproducibility of the PBR measurements, accuracy of the method has been also already evaluated with the use of atomic force microscopy (31493777, 31340868).

*Thank you very much for pointing out these important references. The suggested references have been added and the section rephrased (page 4 Line 69):*

*“The reproducibility of microcirculation measurements of the glycocalyx under the tongue has previously been evaluated (13, 16-20). This was done by use of orthogonal polarization spectroscopy (OPS) in some of the studies (13, 16) and by sidestream dark field (SDF) (17-20) in different patient groups.*

*Furthermore, there has only been one study evaluating the influence of standardised examination conditions for application of the GlycoCheck system however this was only measured at one timepoint after the exposure (17).* *We therefore evaluate both intra- and inter reproducibility as well as day-to-day reproducibility in a group of healthy individuals. Furthermore, the impact of examination conditions in terms of the influence of smoking, side of the tongue measured upon, coffee consumption and intake of a high-calorie meal with repeated measurements over a period of three hours was assessed.”*

Materials & Methods:
Page 6, line 111-113: Could the fact that Days 1 and 2 were up to 7 days apart be responsible for the variation observed? This could be a limitation of the study. Could the observed PBR difference be the result of a change of the systemic glycocalyx? Median days between Days 1 and 2 should be also reported by the authors.

*We have added the number of days between the two visits in the revised Results: “Median (IQR) days between visit 1 and 2 was 3.0 (2.0-6.0).” (line 175, page 9).*

*Interobserver reproducibility was however examined on day 1 where both investigators performed measurements right after each other. This is mentioned under Material and Methods – Study protocol: “we first had a measuring point before intervention by either investigator 1 or 2 according to the randomisation. Then the opposite investigator had a measuring point”.*

*Thus, this would not be an issue for inter-observer reproducibility. The only comparison we have made between days would be for day-to-day variability.*

Moreover, were participants controlled, if they indeed abstained from food, beverages and smoking for eight hours before each visit day? If not, this should be referred in the limitations of the study.

*It is a very valid point that we have no way to control that the participants had abstained from food, beverages and smoking as instructed. We have added this as a limitation (page 17, line 358): “We asked the participants to be fasting before both visits and they had to confirm this before starting the visits, however we could not control this which is a limitation.”*

Page 8, line 157-160: The ICC Models used in every occasion should be clearly reported, as their choice could have a tremendous effect on the results. (Trevethan R. Intraclass correlation coefficients: clearing the air, extending some cautions, and making some requests. Health Serv. Outcome Res Methodol. 2016;17(2):127–43.)

*Thank you very much for making us aware of this problem and a great article for explanation. We have added: “ICC, as implemented in the SAS software, (2,ƙ)” (page 8, line 157).*

Figure 2 – Study design: The study design, as well as how many individuals have been included in each procedure should be better elucidated in the figure.

*Thank you very much for pointing this out. We have updated the Figure 2 and included number of participants.*

Moreover, if 758 measurements have been discarded post-hoc, could that have influenced the results? How have the authors dealt with the missing data?

*It is indeed unfortunate that we had to discard 19% of the measurements. Statistically, we have dealt with the missing data by using mixed model analysis. This we have added under limitations: “Using mixed model analysis accounted for the missing data in the statistical analysis. As data were missing due to a defect equipment (technical difficulties) and not related to clinical characteristics there was no selection bias and data were missing at random.” (page 17, line 351)*

Table 1 & 2: In my opinion, it would be more appropriate to report medians with IQR instead of means with SD. In that way, the influence of possible outliers is being minimized. PBR mean of 3 and PBR mean of 5 should be further reported as means of 3 or 5 measurements.

*As suggested, we have changed reporting of PBR means of 3 or 5 measurements and RBC filling means of 3 or 5 measurements in tables 1 & 2 from mean (SD) to median [IQR].*

Results:

Page 9, line 171: Could the authors please indicate what criteria were used to discard the measurements and what technical issues they faced? (e.g. camera malfunction? Food/drink rests in the optic field? Patients‘ discomfort?)

*We have moved the sentence “due to poor video quality as visually judged by a technician from the GlycoCheck company blinded to all other data in the study” from the limitation to the results section of the study highlighting the way we discarded the measurements.*

*We have clarified that the technical problem we faced was that “one of the four diodes in the camera was broken,” (page 8, line 179).*

Were researchers shown how to avoid pressure artifacts? Did they deliberately try to assess more than one spots in their measurements in order to counterbalance for spatial heterogeneity?

*Thank you for pointing this out. We have added that “Investigators were instructed in handling the GlycoCheck camera and system by the manufacturer. This included an instruction in avoiding using excess pressure to avoid pressure artefacts and in moving the camera to assess more than one spots in order to counterbalance for spatial heterogeneity” (page 6, line 96)*

Tables 2 – 6: Could the authors please specify, what statistical test has been used to calculate the p values?

*In accordance with this helpful comment we have clarified that “Linear mixed-effects models was used as statistical analysis to account for the correlation of repeated measurements within participants.” in the legends to Tables 2-6.*

Discussion:
Page 15, line 316-317: Indeed, more studies are needed to further understand this new technic. However, more than “few studies” have been already conducted. A Pubmed search of “perfused boundary region” shows about 50 conducted studies in different settings.
Page 12, line 241-242: As referred above, more studies than reported have been already conducted to evaluate the reproducibility of the PBR measurements under different conditions (e.g. 31743299, 29444696, 30764695).

*Thank you again for pointing out these important references. The suggested references have been included in the discussion (page 13, line 255):*

*“Reproducibility of the GlycoCheck system has been examined by others. Investigating 70 patients admitted to the emergency room and the intensive care unit Rovas et al. found an excellent intra-observer reproducibility (ICC at 0.77 (CI 0.52-0.89) for PBR and 0.88 (CI 0.74-0.94) for RBC filling) and when comparing measurements performed by nurses and doctors they found a (22). Valerio et al. combined two studies of 50 participants of multi-ethnic origin where two measurements were performed one minute apart and 21 healthy controls with 6 measurements separated by 3 minutes. They found an ICC (CI) of 0.33 (0.08-0.56) for PBR and 0.51 (0.27-0.69) for RBC filling (20). These results are similar to ours (ICC at 0.30 (CI 0.18 to 0.37) for PBR and 0.41 (CI 0.30 to 0.52) for RBC filling). Valerio et al. did not perform series of measurements for mean calculations (20). Weissgerber et al. investigated 13 pregnant women in a day-to-day reliability study. They found that reliability between individual measurements was low with one measurement however with an average of three they primarily found a moderate (ICC 0.50-0.75) to excellent (>0.90) day-to-day reliability though only in a subset of vessel sizes ranging from 9 to 18 µm and only excellent for 12 µm vessels (23). Also investigating ICU patients Bol et al. included 60 participants and in a subset of 20 participants inter-observer reproducibility was investigated. They found an increasing reproducibility with increasing number of measurements reaching 0.80 [IQR 0.68-0.88] and 0.66 [IQR 0.37-0.83] for PBR and 0.90 [IQR 0.84-0.94] and 0.79 [IQR 0.61-0.89] for RBC filling for the two investigators respectively. They found no systematic difference between observers (21).*

*The ICC in our study improved with increasing numbers of measurements, reaching 0.54 (CI 0.38 to 0.62) and 0.65 (CI 0.54 to 0.76) for a mean of 3 measurements for PBR and RBC filling respectively and 0.59 (CI 0.46 to 0.71) and 0.71 (CI 0.61 to 0.82) for a mean of 5 measurements for PBR and RBC filling respectively. Thus, a moderately acceptable level was reached with 5 measurements however only with CI above the threshold for RBC filling. With a mean of both 3 and 5 measurements we were able to detect the effect of interventions. This confirms the reliability of the measurements but stresses the importance of several measurements being performed when using the GlycoCheck system* *where at least three measurements would be advisable.”*

In my opinion, the results are misinterpreted in the discussion section. The authors postulate that a change in PBR after every intervention (meal, coffee, smoking) reflects a change of the systemic endothelial glycocalyx. However, a local effect would be in that case more likely. For example, warm coffee would probably cause locally vasodilatation and the consumption of a meal would probably affect locally the sublingual mucosa. The effect of coffee could have been maybe evaluated if the effect of warm coffee was compared to the possible effect of warm water.

*Thank you for this helpful comment, this could of course be important effects and we have added a paragraph addressing this issue in the Discussion: “As all of the three abovementioned interventions are oral, it should be noted that possibly the observed effects of the interventions are due to a local effect. This does however not change our recommendations to refrain from intake of either of the three at least three hours prior to investigations with the GlycoCheck system.“*

Regarding the inter-observer reproducibility, that fact that the analysis is being performed in different days (up to 7 days), could play a role for the observed PBR differences. A change of the systemic endothelial glycocalyx during that time cannot also be excluded.

*See our comment to the point under material and methods: Indeed, we have up to 7 days between the two visits. Interobserver reproducibility was however examined on day 1 where both investigators performed measurements right after each other. This is mentioned under Material and Methods – Study protocol: “we first had a measuring point before intervention by either investigator 1 or 2 according to the randomisation. Then the opposite investigator had a measuring point”. We have added the number of days in the Results “Median (IQR) days between visit 1 and 2 was 3.0 (2.0-6.0).” (line 175, page 9)*

*Thus, this would not be an issue for inter-observer reproducibility. The only comparison we have made between days would be for day-to-day variability.*

Τhe reported results remain important and help to further standardise the sublingugal glycocalyx measurements and reveal possible interference factors. I would suggest to focus the discussion on the fact, that different factors (e.g. meal, coffee, smoke) seem to intervene significantly with the measurements (locally or systemic cannot be answered by the study design) and that the future researchers need to assess more than one measurements from different positions of the sublingual mucosa to estimate the PBR and RBC filling%.
