# Supplementary material for: Assessment of the sublingual microcirculation with the GlycoCheck system: Reproducibility and examination conditions
Source: PLoS One. 2020 Dec 23;15(12):e0243737. doi: 10.1371/journal.pone.0243737 (PMC7757800; doi:10.1371/journal.pone.0243737)
Supplement: Attachment — Submitted filename: Response to Reviewers MicroREx - 2nd revision.docx [file pone.0243737.s005.docx]

**Answers reviewer comments:**

Reviewer #1:

No Comments

Reviewer #2:


- ICC (2, k) would be the correct ICC form to address inter-rater reliability, when means of measurements are used, but not to check for test-retest reliability. The latter should be assessed with ICC (3, k). (Please check also 27330520 for more details). In case only two single measurements are being used, then ICC (2,1) or ICC (3,1) should be used. Please address this issue, as indeed different ICC forms can lead to tremendous differences.

Thank you for pointing this out. It does indeed make a difference which type of ICC forms are being used. We have addressed this issue by including two forms of ICC calculations in the revised manuscript and updated the manuscript accordantly:

1) Under “Materials and Methods” - “Statistics” we have added: (3,ƙ) where ƙ was 3 and 5 for means of 3 and 5 measurements respectively (Page 8, line 157)
2) This sentence is followed by the suggested reference by Koo et al and a further reference by McGraw et al.
3) Under “Materials and Methods” - “Statistics” we further added: We calculated ICC for means of 3 and 5 measurements respectively by two different methods: First not corrected for increasing number of measurements and secondly with the increasing number of measurements included in the analysis. (Page 9, line 166)
4) Under “Results” - “Measurements” we have recalculated the results and added: When correcting for increasing number of measurements averaged for 3 and 5 measurements respectively: PBR means of 3 = 0.75 (CI 0.66 to 0.84), PBR means of 5 = 0.87 (CI 0.82 to 0.93), RBC filling means of 3 = 0.85 (0.79 to 0.91), and RBC filling means of 5 = 0.93 (CI 0.89 to 0.96). (Page 10-11, line 201)
5) Under “Discussion” we have added:
   a. The ICC was higher when the calculation included the number of measurements. (Page 12, line 245)
   b. The ICC increased markedly when the number of measurements was included in the calculation reaching 0.75 (CI 0.66 to 0.84) and 0.85 (0.79 to 0.91) for means of 3 measurements for PBR and RBC filling, respectively and 0.87 (CI 0.82 to 0.93) and 0.93 (CI 0.89 to 0.96) for means of 5 measurements for PBR and RBC filling, respectively. (Page 14, line 285)
   c. And continuing (addition in red): Thus, a moderately acceptable level was reached with 5 measurements however only with CI above the threshold for RBC filling with results improving when number of measurements was included in the statistical analysis. (Page 14, line 290)

- Could the authors please confirm, that PBR means of 5 (e.g. in Table 3) means, that in every timepoint 5 measurements were made and compared with the PBR means of 5 measurements from the baseline?

We have clarified this point by adding descriptions (here in red) in the legends under Tables 3 to 6, below with the example from Table 3:

Before = the starting value before intervention, mean (standard deviation). 0-180 min shows change over time with no intervention investigating means of 3 and 5 measurements of PBR and RBC filling respectively, compared to means of 3 and 5 measurements before/ at baseline (confidence limits, p-value); N = number of subjects included in the analysis. *p<0.05. Linear mixed-effects model was used as statistical analysis to account for the correlation of repeated measurements within participants. PBR: Perfused Boundary Region; RBC: Red blood cell.

- Line 261 - 262: Please finish the sentence.

Thank you for noticing this. We have finished the sentence with the statement here marked with red:

Reproducibility of the GlycoCheck system has been examined by others. Investigating 70 patients admitted to the emergency room and the intensive care unit Rovas et al. found an excellent intra-observer reproducibility (ICC at 0.77 (CI 0.52-0.89) for PBR and 0.88 (CI 0.74-0.94) for RBC filling) and when comparing measurements performed by nurses and doctors they found a high reproducibility (ICC at 0.75 (CI 0.52-0.87). (Page 13, line 264)

- Rovas et al compared two set of measurements (mean of two measurements vs mean of two further measurements), Valerio et al compared single measurements with each other. Your data, as well as Weissgerber et al and Bol et al confirm the above studies, as they indeed show that the reliability is higher, if means of more than one measurements are being calculated. However, the ICCs of the studies can be only compared directly with cautious, if they are not reported in the original manuscripts. In that case, it might be safer to compare the Bland-Altman plots.

We agree that the form of ICC has not been stated in all the mentioned studies, which makes it difficult to compare. Only the studies by Valerio et al. and Rovas et al. have included calculations of the intra-observer reproducibility using the Bland-Altman plots. The study by Weissgerber et al. has included Bland-Altman plots for the inter-observer reproducibility, but not for the intra-observer reproducibility. and Bol et al. has not applied it at all. As we are comparing 5 single measurements, we cannot apply the methods of Bland-Altman for calculation of the intra-observer variation in our study.

We have added the following precaution to the revised Discussion:

It must be stressed, that only one of the described studies state the form of ICC used. Different ICC forms can lead to significant differences, why comparisons should be performed with caution (reference Koo et al, reference number 25 in manuscript). (Page 14, line 279)

- I would advise the authors, to further tone down regarding the systemic glycocalyx effect of the different interventions and stress that in this study only the local effect was measured. (e.g. Line 305-334).
That does not change the importance of their results, as individuals should refrain from intake before measurements. However, the above passage still gives the false impression, that a direct systemic glycocalyx damage or endothelial dysfunction occurs after coffee consumption or smoking. Although that might indeed be true, it is not supported by the results of this study. To claim the systemic effect of the interventions, either further glycocalyx / endothelial markers should have been measured (e.g. syndecan-1) or the glycocalyx should have been measured on another spot - e.g. ocular.

We have further highlighted the possibility that the effect is local by

1) The following sentence has been moved further up in the manuscript to high lighten the issue: “Measurements with the GlycoCheck devise are assumed to reflect a general status of the systemic glycocalyx. As all three performed interventions are oral it should be noted that the observed effects of the interventions might be due to a transient local effect and not systemic damage to the glycocalyx ~~effect~~. (Page 15, line 311)
2) Revising the sentence after discussing the effect of a high calorie meal and adding the possibility of oral effect (changes in red): “This is in accordance with our findings, ~~using a different measuring technique, we demonstrated~~ indicating a decrease in glycocalyx thickness after a high calorie meal, using a different measuring technique. It must be stressed, that this comparison only holds true if the effect measured with the GlycoCheck camera is systemic and not local, however still suggesting subjects to be fasting for at least 180 minutes before measurement” (Page 16, line 336)
3) In the sentence “Drinking coffee had an immediate negative effect ~~on the glycocalyx~~ on GlycoCheck measurements lasting up till 180 minutes.” we changed “on the glycocalyx” to “on GlycoCheck measurements”. (Page 16, line 341)
4) In the sentence “Others have however demonstrated a ~~similar~~ negative acute effect of coffee consumption though on other markers reflecting endothelial function”. we deleted “similar”. (Page 16, line 344)
5) Adding that “Again, one can only compare when assuming a systemic effect” before concluding that “yet our findings indicate that subjects should refrain from coffee consumption at least for 3 hours prior to the investigation.” (Page 17, line 348)
6) Revising the summarising sentence after we discussed the effects of the interventions to: “The possibility that investigated effect of the three abovementioned interventions is local and not systemic does not change our recommendations to refrain from intake of either of the three at least three hours prior to investigations with the GlycoCheck system”. (Page 17, line 350)

- Table 4 - what does SIGN mean? In case it means significant, the level of significance should be shown as well.

Thank you for pointing this out. This was a mistake and the word SIGN was only present in the manuscript with track changes and not in the “clean” manuscript. It is therefore not corrected in the revised manuscript – just not included.
