# Supplementary material for: Assessment of the sublingual microcirculation with the GlycoCheck system: Reproducibility and examination conditions
Source: PLoS One. 2020 Dec 23;15(12):e0243737. doi: 10.1371/journal.pone.0243737 (PMC7757800; doi:10.1371/journal.pone.0243737)
Supplement: Attachment — Submitted filename: Response to Reviewers MicroREx - 3rd revision.docx [file pone.0243737.s006.docx]

**Answers reviewer comments:**

Reviewer #1:

No Comments

Reviewer #2:

*I would like to thank the authors for successfully addressing my comments and concerns.
I only have a minor suggestion:
Basically, the new ICC analysis shows that multiple measurements (3 to 5) leads to good reproducibility. Therefore, I would suggest integrating in the conclusion the fact that multiple measurements increase the reproducibility of the method dramatically and leads to more robust results.*

Thank you very much for pointing this out. We have highlighted the strengthening of the results with the revised statistical measurements in the appropriate paragraphs in the manuscript. That is the following poins (red is added):

- Abstract, Results, page 2, line 36: ICC was ~~acceptable~~ modest but highly improved by increasing measurements.
- Abstract, Conclusion, page 3, line 42: Measurements with the GlycoCheck system have a moderate reproducibility, but highly increases with multiple measurements and~~, with~~ a small day-to-day variability.
- Main manuscript, Discussion, page 12, line 244: The intra-class correlations showed only a modest reproducibility but ~~even though it was~~ was highly improved with higher numbers of measurements.
- Main manuscript, Discussion, page 13, line 268: These results are similar to ours for single measurements (ICC at 0.30 (CI 0.20 to 0.40) for PBR and 0.45 (CI 0.34 to 0.56) for RBC filling).
- Main manuscript, Discussion, page 14, line 288: Thus, a moderately acceptable level was reached with 5 measurements however only with CI above the threshold for RBC filling with results greatly improving when number of measurements was included in the statistical analysis.
- Main manuscript, Discussion, page 18, line 382: In conclusion, we found measurements of glycocalyx and microvascular perfusion with the GlycoCheck system to be with a moderate reproducibility, but markedly improved with multiple measurements, and measures affected by examination conditions.
